# Memory of pain in adults: a protocol for systematic review and meta-analysis

**DOI:** 10.1186/s13643-019-1115-4

**Published:** 2019-08-13

**Authors:** Wacław M. Adamczyk, Dominika Farley, Karolina Wiercioch-Kuzianik, Elżbieta A. Bajcar, Ewa Buglewicz, Jakub Nastaj, Aleksandra Gruszka, Przemysław Bąbel

**Affiliations:** 1 Pain Research Group, Institute of Psychology, Jagiellonian University, ul. Ingardena 6, 30-060 Kraków, Poland; 2 Laboratory of Pain Research, The Jerzy Kukuczka Academy of Physical Education, Katowice, Poland

**Keywords:** Acute pain, Chronic pain, Memory of pain, Recollection, Remembrance

## Abstract

**Background:**

The way pain is remembered and reported can affect medical decisions taken by patients and health-care professionals. Memory of pain has been investigated extensively for the past few decades; however, the results of previous studies are highly variable, indicating that the recollection of pain can be accurate, overestimated or underestimated. It is therefore difficult to conclude how well pain is remembered. The aim of this systematic review and meta-analysis is to summarize research findings on memory of pain in healthy adults and patients suffering from acute and chronic pain.

**Methods:**

The systematic review will be performed by searching for articles indexed in the following databases: PubMed, MEDLINE, PsycINFO, Web of Science, ScienceDirect, PsycARTICLES, Scopus and Academic Search Complete. Studies will be included if (1) they investigated healthy adults or patients with any acute or chronic pain condition and if (2) they assessed experienced pain (pain intensity and/or pain unpleasantness) and its recollection. No restrictions related to the date of publication and recall delay will be applied. Studies will be screened for eligibility and risk of bias by two independent assessors. The risk of bias will be assessed by a modified Downs and Black checklist. A narrative synthesis will be performed in the first stage; in the second stage, the results of studies with comparable designs will be pooled in meta-analytical syntheses.

**Discussion:**

The question of whether pain is remembered accurately is crucial for valid pain diagnosis, effective treatment and prognosis. So far, a number of studies on memory of pain have been conducted; however, a definitive conclusion on whether memory of pain is accurate is still lacking. In this systematic review and meta-analysis, available data will be pooled together to further inform research and clinical practice.

**Systematic review registration:**

PROSPERO CRD42018093523

**Electronic supplementary material:**

The online version of this article (10.1186/s13643-019-1115-4) contains supplementary material, which is available to authorized users.

## Background

The lack of agreement on how well pain intensity is remembered poses a serious problem for clinical practice. Reports of pain experiences provided by patients form the basis for diagnoses and treatments [1, 2]. Pain recollections may also influence the willingness of patients to undergo future medical procedures [3, 4]. Although memory of pain has been investigated extensively for the few last decades (for reviews see [5, 6]), the results of previous studies are highly variable. Several studies have shown that pain may be remembered accurately [7–15], but there is also evidence indicating that people tend to overestimate [16–19] or underestimate their recollections of pain [20–24]. Thus, a definitive answer to the question of whether pain is remembered accurately is still lacking.

Many factors have been found to influence the accuracy of memory of pain (for reviews see [5, 6]). Two of them are believed to be of special importance: (1) type of pain, which refers to the origin of pain and its duration (i.e. chronic pain, naturally occurring acute pain and experimentally induced acute pain), and (2) recall delay, which refers to the interval between the experience of pain and its recall. For practitioners, these are the first and most accessible pieces of information concerning pain experienced by the patient; however, the effect of these two factors has rarely been investigated in a single study. By using a meta-analytic approach, the results of studies differing in terms of recall delay and type of pain could be compared systematically in order to elucidate the effects of recall delay and type of pain on memory of pain.

It seems the type of pain can influence the distortion of memory of the pain. Previous studies have shown that chronic pain can be overestimated [16, 25], while acute pain can be either overestimated [19, 26–28] or underestimated [22–24, 29]; however, in other studies, there was no effect of the type of pain on its recall [12–15]. To the best of our knowledge, only one study aimed to investigate the effect of the source of pain (e.g. surgery vs. labor) on its recall [30]. A meta-analytic approach may be successfully used to compare the results of previous studies on different types of pain in order to find out whether memory of pain differs with regard to the type of pain. More studies focused on the role of recall delay. Generally, lower accuracy of recall in the case of longer reporting periods has been observed in a few previous studies [21, 23, 31–33]. At the same time, other studies failed to find an effect of recall delay on memory of pain [7–10, 20]. Again, a meta-analytic approach may help in comparing the results of previous studies in which different recall delays were applied to find out whether memory of pain depends on recall delay.

We will attempt to conduct a systematic review and meta-analysis of previous studies on memory of pain in order to answer the following questions:

- Is pain remembered accurately in adults?
- Does the type of pain affect memory of pain in adults?
- Does recall delay affect memory of pain in adults?

## Methods

This review protocol was designed a priori according to the Preferred Reporting Items for Systematic Review and Meta-Analyses Protocols (PRISMA-P checklist form) guideline [34]. The systematic review protocol followed the recommendations on data searching and data processing described in the Cochrane Handbook for Systematic Reviews [35]. The review protocol was registered in the PROSPERO database under number CRD42018093523. Before starting the review procedures, the authors will be involved in a training session to practice the procedures required to perform a systematic review and meta-analysis.

### Training session

The authors will be divided into teams of two researchers. Each team will be involved in a different area of the research: (1) searching, identification and selection of studies; (2) assessment of the risk of bias; (3) data extraction; and (4) data synthesis. In order to ensure consistency of assessment strategies between assessors, each team will participate in a trial search (the first team) and assessment of 10 randomly chosen articles (remaining teams). Thus, the search and assessment criteria will be discussed and standardized before the beginning of the actual research. Furthermore, the agreement between both authors will be evaluated regarding the selection of studies, risk of bias assessment and data extraction.

### Search strategy for identification of studies

The PICOS framework will be used to develop a search strategy [34]. The chosen search terms will refer to the studied population of adult humans (P), experienced pain as a comparator (C) and memory of pain as an outcome (O). Intervention (I) will be omitted because it does not apply to our research questions; study type (S) will be omitted in order to avoid the exclusion of relevant studies. In the review process, we will consider and evaluate studies published in English only. There will be no restrictions regarding the timeframe of published articles. Medical subject heading (MeSH) terms and natural language expressions will be searched for in electronic databases.

### Search terms and phrases

The search strategy that will be used for the PubMed database is listed below. Studies with phrases referring to animal models will be excluded only when a significant number of records is found in a given database.

1. pain*
2. memory
3. recall*
4. remember*
5. retrospective*
6. #2 OR #3 OR #4
7. child*
8. animal
9. rat
10. mouse
11. mice
12. #6 OR #7 OR #8 OR #9 OR #10
13. #1 AND #5 NOT #11

### Databases

Eight databases will be searched for relevant articles including:

1. PubMed
2. MEDLINE
3. PsycINFO
4. Web of Science
5. ScienceDirect
6. PsycARTICLES
7. Scopus
8. Academic Search Complete

### Searching other resources

In order to extend the scope of the review, three additional sources of data will be searched. First of all, the reference lists of identified articles and key review articles will be screened for other relevant articles. Secondly, a review of online-available contents of five pain-related journals will be performed electronically: *Pain*, *The Journal of Pain*, *European Journal of Pain*, *Pain Medicine* and *The Clinical Journal of Pain*. Moreover, experts on memory of pain will be contacted and asked to refer us to any relevant studies consistent with the topic of this systematic review and meta-analysis that might have been omitted.

### Selection of relevant studies

Only studies which meet the aforementioned criteria for inclusion and exclusion presented in Table 1 will be included in the systematic review. Only studies investigating adult humans will be selected. Only those studies which contain a procedure wherein pain is assessed at a minimum of two separate points in time will be included. The assessment should be performed by means of a self-report of experienced pain using a scale that gives a numeric, countable outcome (e.g., VAS, NRS, VRS) of pain intensity (sensory dimension of pain) and/or pain unpleasantness (affective dimension of pain). The scales may be part of other assessment tools, but they should comply with the aforementioned criteria in order to be taken into account. The assessment of actual pain must be performed by the participant while experiencing pain or shortly thereafter (first time point). The assessment of memory of pain must refer to the past experience of actual pain (second time point); however, if memory of pain refers to averaged pain experienced at multiple time points (e.g. diary studies), the study will be excluded.

**Table 1:** Selection criteria for systematic review

Item	Inclusion criteria	Exclusion criteria
P	- Studies on adult humans - Patients with chronic or acute pain and pain-free healthy volunteers	- Studies including children - Studies on animals
I	- N/A	- Clinical trials without control group (no intervention group)
C	- Original pain rating (experienced pain intensity and/or pain unpleasantness)	- N/A
O	- Pain rating after delay, i.e. representing memory of pain - The assessment should be performed on a scale (e.g., VAS, NRS, VRS) that gives a numeric, countable outcome	- Studies in which different dimensions of experienced and recalled pain were assessed - Studies in which experienced and recalled pain were assessed on different scales/tools
S	- N/A	- Review articles, conference communications, case studies, letters, editorials, diary studies

*P* participants, *I* intervention, *C* comparison, *O* outcome, *S* study type, *N/A* not applicable, *VAS* visual analogue scale, *NRS* numeric rating scale, *VRS* verbal rating scale

We will exclude studies in which different dimensions of experienced and recalled pain were assessed (e.g. pain intensity assessed at time point one, but memory of pain unpleasantness assessed at time point two) or in which different scales were applied to assess experienced and recalled pain (e.g. NRS at one point, but VAS at the second time point). Generally, only studies with no additional manipulation that is intended to change the memory will be included. However, if data collected from a group of participants who were not subject to any manipulation of pain recall is available in this kind of study (e.g. a control group), that part of the data will be included in our review. We shall take into consideration three separate kinds of research: one focused on experimentally induced pain, one focused on chronic pain and one focused on acute pain. In the first kind of research, pain is induced by various experimental interventions such as electrocutaneous, thermal or pressure stimulation. In the second and third kind, pain occurs as a result of an injury, a medical intervention such as surgery (e.g. Caesarean section), autonomous diseases (e.g. osteoarthritis, lower back pain) or is non-specific without an underlying pathological cause. Studies will be classified as investigating chronic pain if its duration is reported as longer than 3 months [36]. In other cases, studies will be classified as examining acute pain.

### Data collection and analysis

#### The procedure of studies selection

Two stages of study selection will be performed: preliminary and final. The preliminary study selection will involve screening by two independent raters of the titles and abstracts of the identified articles/publications according to the eligibility criteria. Subsequently, in the final stage the full-text articles of the remaining studies will be reviewed using the same inclusion/exclusion criteria (Table 1). References will be managed with EndNote X8 software (Clarivate Analytics, Philadelphia, USA). The agreement between the two assessors will be expressed using Cohen’s kappa coefficient, a statistical coefficient which is commonly used for nominal/categorical data [37]. In the case of a disagreement between the two assessors that cannot be resolved through discussion, a third independent expert will be involved and will decide whether the publication will be included in the review process or not. This procedure follows the recommendations described in Chapter 7 of the Cochrane Handbook [35]. The selection process will be documented in a flowchart recommended within the PRISMA statement [38].

#### Risk of bias assessment tool

All studies which meet the inclusion/exclusion criteria will be carefully assessed with regard to the potential risk of bias by two independent assessors using the checklist developed by Downs and Black [39] and recommended in the *Cochrane Handbook for Systematic Reviews of Interventions* [35]. The checklist has been used previously in meta-analyses of pain-related studies, and a modified form has also been used [40–42]. In our case, its contents were modified in order to apply to the type of studies which are most likely to meet the inclusion/exclusion criteria, as well as to include clinical trials and experimental studies. Specifically, questions relating to the intervention used were removed, since that type of medical intervention lies beyond the scope of our interest and does not apply to experimental studies. Some questions have been modified to reflect methodological issues crucial for memory of pain studies (see Additional file 1: Table S1). Moreover, in order to create a unified power criterion for all the studies (question 27 in the original checklist), an a priori power calculation will be performed based on the results of two studies that used similar methodology to investigate memory of pain [20, 21]. The results of this calculation will serve as a criterion by which to judge other studies (Downs and Black checklist [39], question 27).

A detailed list of included and modified questions is presented in Additional file 1: Table S1. Cohen’s kappa coefficient will be used to calculate the level of agreement between assessors. In the case of a disagreement, the assessors will discuss the reasons for their judgement and if the difference in opinion remains unresolved, a third assessor will arbitrate. The final risk of bias judgement will be presented in a table.

#### Data extraction

Data will be extracted from all included studies by two review co-authors independently; the results obtained by each will be compared to ensure accuracy and the data will be included only if both reviewers obtain the same result. Any disagreements will be discussed between the assessors; if a consensus cannot be reached, a third assessor will assist in making the final decision. Data will be extracted using a pre-designed data extraction sheet. The following data will be extracted from studies: sample size, age, gender, general type of pain (acute, chronic, experimentally induced), source of pain (e.g. labor, surgery), nature of noxious stimuli (e.g. thermal, capsaicin-induced), characteristics of pain stimulation (e.g. mA, °C), duration of pain, dimension of pain (e.g. sensory, affective), type of pain assessment tools (e.g., NRS, VAS, VRS) and scale anchors, methods of collecting the pain assessments (e.g. paper and pencil, telephone, online survey), recall delay between the experience of pain and its recollection, number of assessments of the pain experience and memory of pain, type of pain assessment (e.g. average, maximum, minimum, end) and data from the assessment of the pain experience and its recall (e.g., means and standard deviations). Extracted data will be used to characterize and describe the methodology of the included studies.

In the case of any disparity in the extracted data, final tables presenting the study characteristics will be created as a result of a discussion between the co-authors of the review. Study authors will be contacted by email in order to obtain the missing information.

#### Data synthesis

All identified studies will be included in the narrative synthesis and presented in separate tables. The results will be combined in the meta-analysis directed at answering the primary question of whether memory of pain is distorted or not. However, only studies in which both the experienced and recalled pain was assessed using the same scale will be pooled into meta-analytical synthesis using random effect models. If randomized controlled trials are included in the review, only the data obtained from groups that were not exposed to any intervention intending to change memory of pain will be entered into the meta-analysis.

Means and standard deviations (SDs) will be combined; if standard errors or confidence intervals are reported in the studies, the appropriate transformation into means and SDs will be carried out. Mean values and standard deviations of experienced and recalled pain weighted by the sample size will be included in the analysis and presented as a standardized mean difference in order to overcome the differences in pain scales used in different studies.

The analyses will be divided into three stages: (1) main analyses, (2) sensitivity analyses and (3) subgroup analyses. For the main analyses, all included studies assessing pain intensity and pain unpleasantness will be combined, respectively. Then, sensitivity analyses will be performed with exclusion of studies in which the quantitative dimension of pain is different during recall (e.g., maximum pain remembered) than in the initial phase, when the experienced pain assessment was collected (e.g. momentary pain). If sensitivity analyses show significantly different results after exclusion of these studies, the following third stage (subgroup analyses) will be carried out without these studies. The third stage will be performed on subgroups created based on the type of pain being studied (acute pain, experimental pain, chronic pain) and the recall delay applied (< 24 h, 24 h < 1 week, 1 week < 1 month, >1 month). Publication bias will be evaluated through visual inspection of funnel plots.

Any further quantitative syntheses and deviations from this plan will be treated as explorative and reported as such. All calculations will be performed using the Review Manager (RevMan software version 5.3) provided by the Cochrane Collaboration (Copenhagen, The Nordic Cochrane Centre, The Cochrane Collaboration).

### Further steps

The review will be prepared for publication in a scientific journal that accepts articles in the field of pain science or medicine, such as *Pain* or *The Journal of Pain.* If any modification to this protocol is introduced, it will be clearly explained in the manuscript body.

## Discussion

Currently, no clear consensus exists on whether or not people remember pain accurately and which factors might let us predict with relative certainty how pain will be remembered. Elucidating this problem could have a profound impact on interpretation of patients’ pain in research and clinical practice. Reports of pain experienced in the past are taken into account by medical professionals when diagnosing patients and deciding on appropriate treatments [1, 2]; they also form the basis of the assessment of such treatment. Memory of past pain influences decisions concerning whether to engage in future painful experiences, e.g. undergoing painful medical examinations [3, 4] and how painful those experiences will be [18]; moreover, it can also influence the development of chronic pain [43, 44]. Previous research has found that when pain was recalled as having greater intensity than the actual pain experience, patients reported greater pain relief after active and placebo treatments [28, 31, 45], but those who reported complete pain relief during the pain experience were unable to recollect that pain relief at a 6-month follow-up [26]. Thus, the accuracy of memory of pain is an important problem in clinical practice. We hope that the results of this meta-analysis will help to better understand and interpret pain reported by patients.

## Additional files

Additional file 1: **Table S1.** Risk of bias assessment checklist based on Black and Downs Scale [39]*. (DOCX 19 kb)

## Data Availability

Not applicable.
