# Supplementary material for: Memory of pain in adults: a protocol for systematic review and meta-analysis
Source: Syst Rev. 2019 Aug 13;8:201. doi: 10.1186/s13643-019-1115-4 (PMC6693173; doi:10.1186/s13643-019-1115-4)
Supplement: Supplementary file 1 — Table S1. Risk of bias assessment checklist based on Black and Downs Scale [39]*. (DOCX 19 kb) [file 13643_2019_1115_MOESM1_ESM.docx]

**Table S1. Risk of bias assessment checklist based on Black and Downs Scale [39]*.**

| **Original question number** | **Original question (answer-points)** | **How was it modified and why?** | |
| --- | --- | --- | --- |
| 1 | Is the hypothesis/aim/objective of the study clearly described? (yes=1; no=0) | left without modification | |
| 2 | Are the main outcomes to be measured clearly described in the Introduction or Methods section? (yes=1; 0=no) | left without modification | |
| 3 | Are the characteristics of the patients included in the study clearly described? (yes=1; no=0) | left without modification | |
| 4 | Are the interventions of interest clearly described? (yes=1; no=0) | modified in order to comply with the subject of our systematic review and meta-analysis: Were the settings of obtained comparator (actual pain rating) and outcome (recalled pain rating) similar? (similar settings=1; different settings=0; unable to determine=0) | |
| 5 | Are the distributions of principal confounders in each group of subjects to be compared clearly described? (yes=2; partially=1; no=0) | yes=2 if more than 1 additional characteristic apart from sex and age of participants and characteristics relevant to memory of pain (e.g. type of pain, pain experienced during recall, etc.) is given; partially=1 if 1 additional characteristic apart from sex and age of participants and no characteristics relevant to memory of pain is given; no=0 if only age and sex of participants is given, or less | |
| 6 | Are the main findings of the study clearly described? (yes=1 if simple outcome data is reported in the text or on a graph; no=0) | left without modification | |
| 7 | Does the study provide estimates of the random variability in the data for the main outcomes? (yes=1; no=0) | left without modification | |
| 8 | Have all important adverse events that may be a consequence of the intervention been reported? (yes=1; no=0) | excluded: medical or pharmacological treatments are not the subject of the meta-analysis, which also includes experimental studies without any such treatments; therefore, this question does not apply | |
| 9 | Have the characteristics of patients lost to follow-up been described? (yes=1; no=0) | left without modification | |
| 10 | Have actual probability values been reported (e.g.0.035 rather than <0.05) for the main outcomes except where the probability value is less than 0.001 (yes=1; no=0) | left without modification | |
| 11 | Were the subjects asked to participate in the study representative of the entire population from which they were recruited? (yes=1; no=0; unable to determine=0) | left without modification | |
| 12 | Were those subjects who were prepared to participate representative of the entire population from which they were recruited? (yes=1; no=0; unable to determine=0) | left without modification | |
| 13 | Were the staff, places, and facilities where the patients were treated representative of the treatment the majority of patients receive? (yes=1; no=0; unable to determine=0) | excluded: it applies to clinical trials only | |
| 14 | Was an attempt made to blind study subjects to the intervention they received? (yes=1; no=0; unable to determine=0) | modified in order to comply with the subject of our systematic review and meta-analysis: Was an attempt made to blind study subjects to the fact that they would be asked to recall the intensity of their pain at a later time? Punctation left without modification | |
| 15 | Was an attempt made to blind those measuring the main outcomes of the intervention? (yes=1; no=0; unable to determine=0) | specified: “those measuring the main outcomes” included examiners | |
| 16 | If any of the results of the study were based on “data dredging”, was this made clear? (yes=1; no=0; unable to determine=0) | modified: If any of the results of the study were not based on “data dredging”? (yes=1; no=0; unable to determine=0) | |
| 17 | In trials and cohort studies, do the analyses adjust for different lengths of follow-up of patients, or in case-control studies is the time period between the intervention and outcome the same for cases and controls? (yes=1; no=0; unable to determine=0) | excluded: medical or pharmacological treatments are not the subject of the meta-analysis, which also includes experimental studies without any such treatments; therefore, this question does not apply; different lengths of time between pain measurement and recall taken into account. | |
| 18 | Were the statistical tests used to assess the main outcomes appropriate? (yes=1; no=0; unable to determine=0) | left without modification | |
| 19 | Was compliance with the intervention/s reliable? (yes=1; no=0; unable to determine=0) | excluded: medical or pharmacological treatments are not the subject of the meta-analysis, which also includes experimental studies without any such treatments; therefore, this question does not apply | |
| 20 | Were the main outcome measures used accurate (valid and reliable)? (yes=1; no=0; unable to determine=0) | left without modification |  |
| 21 | Were the patients in different intervention groups (trials and cohort studies) or were the cases and controls (case-control studies) recruited from the same population? (yes=1; no=0; unable to determine=0) | excluded: medical or pharmacological treatments are not the subject of the meta-analysis, which also includes experimental studies without any such treatments; therefore, this question does not apply |  |
| 22 | Were study subjects in different intervention groups (trials and cohort studies) or were the cases and controls (case-control studies) recruited over the same period of time? (yes=1; no=0; unable to determine=0) | excluded: medical or pharmacological treatments are not the subject of the meta-analysis, which also includes experimental studies without any such treatments; therefore, this question does not apply |  |
| 23 | Were study subjects randomized to intervention groups? (yes=1; no=0; unable to determine=0) | excluded: medical or pharmacological treatments are not the subject of the meta-analysis, which also includes experimental studies where random allocation into groups is a standard procedure |  |
| 24 | Was the randomized intervention assignment concealed from both patients and health care staff until recruitment was complete and irrevocable? (yes=1; no=0; unable to determine=0) | excluded: medical or pharmacological treatments are not the subject of the meta-analysis, which also includes experimental studies without any such treatments; therefore, this question does not apply |  |
| 25 | Was there adequate adjustment for confounding in the analyses from which the main findings were drawn? (yes=1; no=0; unable to determine=0) | specified: yes=1 (distribution of known confounders described and taken into account in the analyses); no=0 (confounders ignored or observed but not analyzed); unable to determine=0 |  |
| 26 | Were losses of patients to follow-up taken into account? (yes=1; no=0; unable to determine=0) | left without modification |  |
| 27 | Did the study have sufficient power to detect a clinically important effect where the probability value for a difference being due to chance is less than 5%? | specified: studies with power (calculation based on the results of two studies that used similar methodology of memory of pain investigation): <75% - 0; 76%-80% - 1; 81%-85% - 2; 86%-90% - 3; 91%-95% - 4; >96% - 5 |  |

*The original Black & Downs Scale is comprised of 27 questions which were described in detail by the authors [39]. For the purpose of our meta-analysis, some questions were omitted.
